# The cuticle modulates ultraviolet reflectance of avian eggshells

**DOI:** 10.1242/bio.012211

**Published:** 2015-05-11

**Authors:** Daphne C. Fecheyr-Lippens, Branislav Igic, Liliana D'Alba, Daniel Hanley, Aida Verdes, Mande Holford, Geoffrey I. N. Waterhouse, Tomas Grim, Mark E. Hauber, Matthew D. Shawkey

**Affiliations:** 1 Department of Biology, University of Akron, Akron, OH 44325, USA; 2 Department of Zoology and Laboratory of Ornithology, Palacký University, Olomouc 771 46, Czech Republic; 3 Department of Chemistry, Hunter College and the Graduate Center, City University of New York, New York, NY 10021, USA; 4 School of Chemical Sciences, University of Auckland, Auckland 1142, New Zealand; 5 Department of Psychology, Hunter College and the Graduate Center, City University of New York, New York, NY 10065, USA

**Keywords:** Avian eggshells, Cuticle, Light modulation, Ultraviolet reflectance, Biomimicry

## Abstract

Avian eggshells are variedly coloured, yet only two pigments, biliverdin and protoporphyrin IX, are known to contribute to the dramatic diversity of their colours. By contrast, the contributions of structural or other chemical components of the eggshell are poorly understood. For example, unpigmented eggshells, which appear white to the human eye, vary in their ultraviolet (UV) reflectance, which may be detectable by birds. We investigated the proximate mechanisms for the variation in UV-reflectance of unpigmented bird eggshells using spectrophotometry, electron microscopy, chemical analyses, and experimental manipulations. We specifically tested how UV-reflectance is affected by the eggshell cuticle, the outermost layer of most avian eggshells. The chemical dissolution of the outer eggshell layers, including the cuticle, increased UV-reflectance for only eggshells that contained a cuticle. Our findings demonstrate that the outer eggshell layers, including the cuticle, absorb UV-light, probably because they contain higher levels of organic components and other chemicals, such as calcium phosphates, compared to the predominantly calcite-based eggshell matrix. These data highlight the need to examine factors other than the known pigments in studies of avian eggshell colour.

## INTRODUCTION

Understanding the proximate causes of variation in morphological traits like colour is critical to understanding their functions and evolution (Hill and McGraw, 2006). Eggshell coloration may serve several roles, including camouflage (Merilaita and Lind, 2005), sexual selection (Moreno and Osorno, 2003), or host-parasite egg mimicry and rejection (Yang et al., 2013). A recent study further suggested that colour produced by pigments modulates the amount of beneficial vs. harmful UV-light reaching the embryo by acting as an absorbing barrier (Maurer et al., 2015). However, many eggshells lack pigmentation (Hauber, 2014) and the mechanism by which they attenuate ultraviolet light is unknown (Kilner, 2006). Studying the proximate basis of egg coloration may also help provide inspiration for applied systems, including the development of biomimetic materials by identifying important factors that contribute to light modulation (Yoo et al., 2009; Li et al., 2010). Colours in nature can be produced by pigments, nanostructured architectures (generating structural colour), or a combination of both (Parker, 2000; Sun et al., 2013). Whereas pigments produce colour through the absorbance of light at specific wavelengths, structural colours are produced by selective reflectance, scattering or diffraction of light by nanostructured biological materials (Kinoshita et al., 2008; Srinivasarao, 1999).

Little is known about the mechanisms that generate eggshell coloration. Currently, only two classes of tetrapyrrole pigments (biliverdin and protoporphyrin IX) are considered to influence eggshell coloration of most bird species (Kennedy and Vevers, 1976). However, recent studies have shown that eggshell coloration of a number of different species cannot be explained solely by variation in biliverdin and protoporphyrin concentrations (Cassey et al., 2012a; Igic et al., 2012), suggesting that other mechanisms may contribute to the appearance of eggshells. Indeed, in addition to the two tetrapyrrole pigments avian eggshells consist of numerous other compounds that may selectively absorb light or modify the absorption properties of the two pigments.

In addition to pigments, eggshell proteins or nanostructures could contribute to eggshell coloration by either selectively absorbing certain wavelengths or enhancing light reflectance, respectively. Eggshells consists of about 4% organic and 96% inorganic material, the latter of which 98% is calcium carbonate, and the remainder includes calcium phosphates and metal ions (Hamilton, 1986). Furthermore, the external eggshell surface of most avian species is covered by a cuticle, a non-crystalized layer that can vary in thickness and consist of proteins, polysaccharides, lipids, calcium carbonate, and calcium phosphates (Kusuda et al., 2011; Mikhailov, 1997; Wedral et al., 1974). Aromatic amino acids of proteins (Holiday, 1936) and calcium phosphates (Bogrekci and Lee, 2004; Holzmann et al., 2009) also have distinctive absorption spectra compared to calcite and the two tetrapyrrole pigments. Both groups of molecules absorb maximally in the (near) UV-range, and are common constituents of eggshells (Hincke et al., 1992; Sparks, 1994). Moreover, the nanostructural organisation of calcium carbonate can produce structural colour [e.g. nacre (Grégoire, 1957; Bonderer et al., 2008; Finnemore, 2012)]. Critically, the eggshell cuticle differs both in composition and structure from the underlying crystalized eggshell (Baker and Balch, 1962; Kusuda et al., 2011) and therefore may differentially affect light modulation. Indeed, it has been shown that an extremely smooth cuticle produces glossiness and iridescence in tinamou eggs (Igic et al., 2015).

Here, we investigated mechanisms underlying colour variation of immaculate, white avian eggshells. We specifically examined how the eggshell cuticle contributes to coloration. To do this, we experimentally removed the outer layers of immaculate, white eggshells of four species: chicken (*Gallus gallus*), Australian brushturkey (*Alectura lathami*), king pigeon (*Columba livia domestica*), and budgerigar (*Melopsittacus undulatus*). If the cuticle contributes to eggshell coloration, we predicted that its removal would cause a larger colour change in eggshells with cuticles compared to those without. We then used scanning electron microscopy, X-ray photoelectron spectroscopy, and chemical extractions to investigate if nanostructural features or chemical composition explain the observed patterns of coloration and its change following experimental manipulation.

## RESULTS

Ultra High Performance Liquid Chromatography (UHPLC) and Mass Spectrophotometry (MS) confirmed that none of the eggshells of the four species (chicken, brushturkey, pigeon, and budgerigar) contained any detectable concentrations of protoporphyrin or biliverdin, whereas these pigments were detected in our positive controls (supplementary material Fig. S1).

**Fig. 1.**
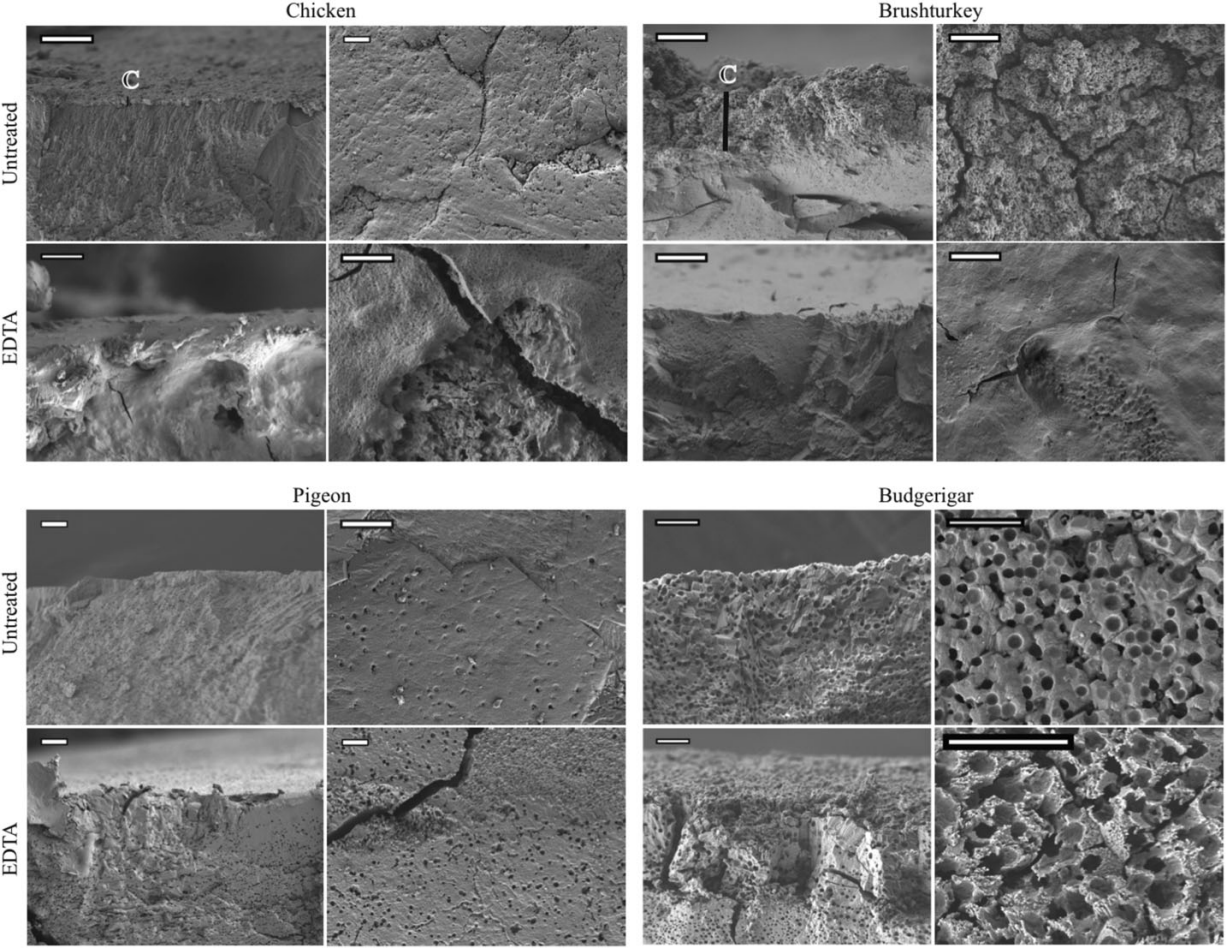
**SEM images showing the different eggshell morphologies for untreated and EDTA treated eggs.** The EDTA treatment durations are 90 min for chicken, brushturkey, pigeon, and 30 min for budgerigar. First and third column are cross-sections, second and fourth column are topview images. C=Cuticle layer. Scale bars are 10 µm.

Untreated eggs of the four species differed in overall structure, thickness and presence of cuticle (Fig. 1; Table 1). Chicken eggs were covered by a thin smooth cuticle that contained nanospheres with a mean diameter of 151.4±5.2 nm (*n*=40, s.e.m.). Brushturkey eggshells had a distinct cuticle composed of nanospheres with a mean diameter of 307.8±13.1 nm (*n*=40, s.e.m.). Pigeon eggshells had a smooth surface with some pores, and cross-section images for one of the eggs showed a structure resembling a very thin cuticle (supplementary material Fig. S2). Budgerigar eggshells lacked a cuticle, and the vesicles of the organic matrix were visible on the surface as pores with a diameter varying between 1–2 μm in diameter (Fig. 1).

**Table 1.**
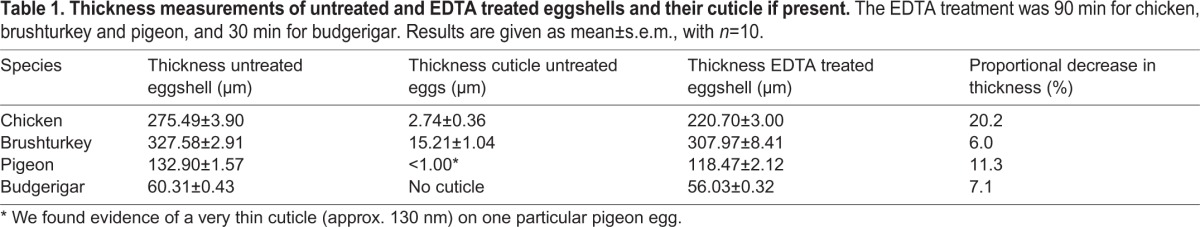
**Thickness measurements of untreated and EDTA treated eggshells and their cuticle if present.** The EDTA treatment was 90 min for chicken, brushturkey and pigeon, and 30 min for budgerigar. Results are given as mean±s.e.m., with *n*=10.

Sequential treatment with ethylenediaminetetraacetic acid (EDTA) gradually removed the outer layers of all four species’ eggshells, but had differential effects on their structure (Fig. 1; supplementary material Fig. S3) and decrease in thickness (Table 1). After 30 min of EDTA treatment, the nanospheres of chicken eggshell cuticle were removed (supplementary material Fig. S3), whereas after 90 min of EDTA treatment, the cuticle was fully removed along with a portion of the underlying palisade layer (Fig. 1). After 30 min of EDTA treatment, only a few nanospheres were still present on the brushturkey eggshell (supplementary material Fig. S3), and after 90 min of EDTA treatment, parts of the underlying palisade layer became visible and removal of the cuticle was confirmed in the cross-section image (Fig. 1). After sequential EDTA treatment, the vesicles of the pigeon eggshell became gradually more distinct as deeper pores according to the time of the treatment (Fig. 1; supplementary material Fig. S3). After 30 min of EDTA treatment, the holes on the budgerigar eggshell were still visible, however, the surface became much rougher and pockmarked (Fig. 1).

**Fig. 2.**
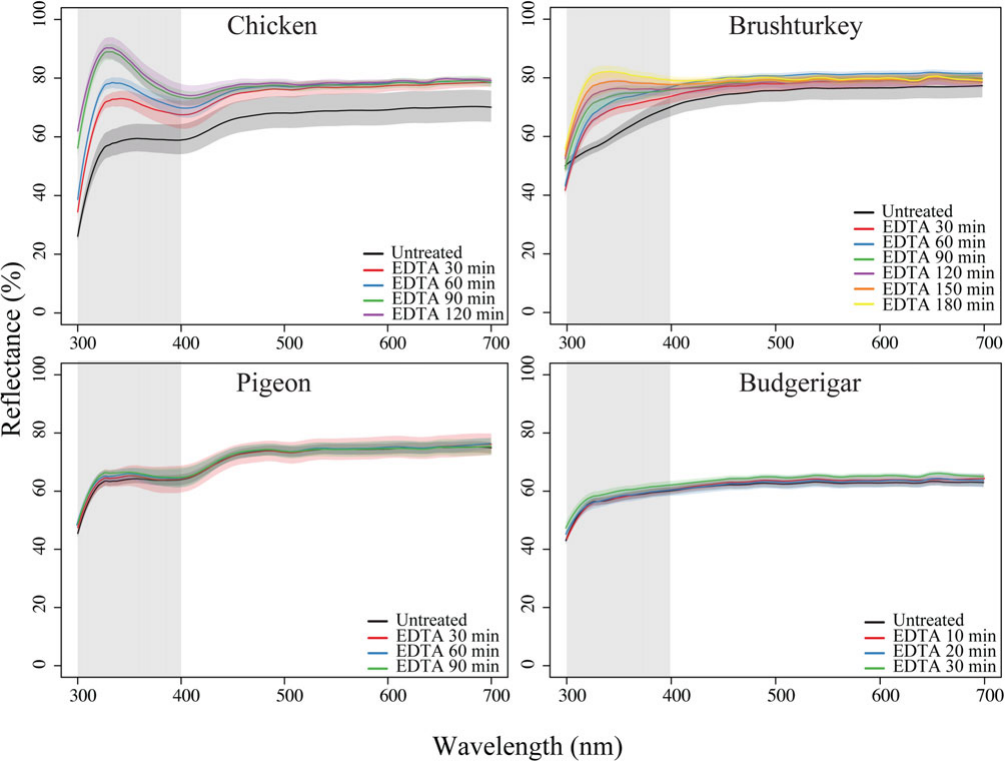
**The effect of EDTA treatment on diffuse reflectance of white-coloured eggshells from chicken, brushturkey, pigeon and budgerigar.** Durations for EDTA treatment were different for budgerigar, as the eggshells were very fragile. Plotted lines are group mean spectra (*n*=3) with shaded areas representing the standard error. Grey area represents the UV-region, highlighting differences in reflectance.

**Fig. 3.**
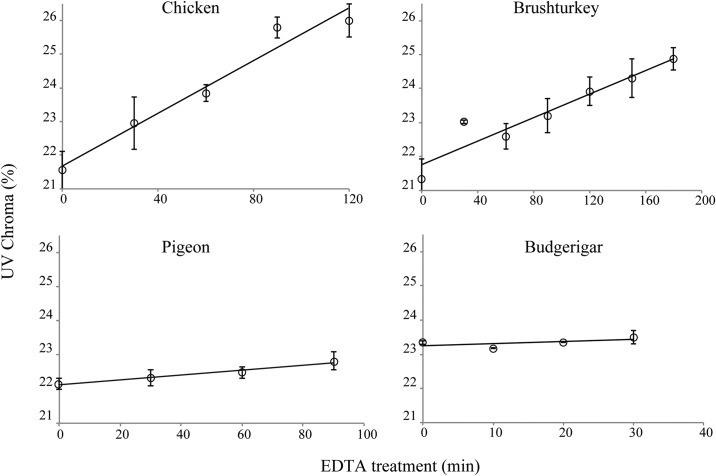
**UV-chroma as a function of the duration of EDTA treatment.** The data are presented as means±s.e.m. Note that the x-axis scales are different for each species.

Gradual removal of the outer layers (including the cuticle if present) resulted in a significant increase in UV-chroma for chicken and brushturkey eggs. With increasing chemical etching of the outer layers, UV-chroma increased for chicken (*F*_1,11_=103.7, *P*<0.001), brushturkey (*F*_1,17_=62.0, *P*<0.001), and pigeon (*F*_1,8_=11.6, *P*<0.01), but not for budgerigar (*F*_1,8_=1.8, *P*=0.22) (Figs 2,3; Table 2).

**Table 2.**
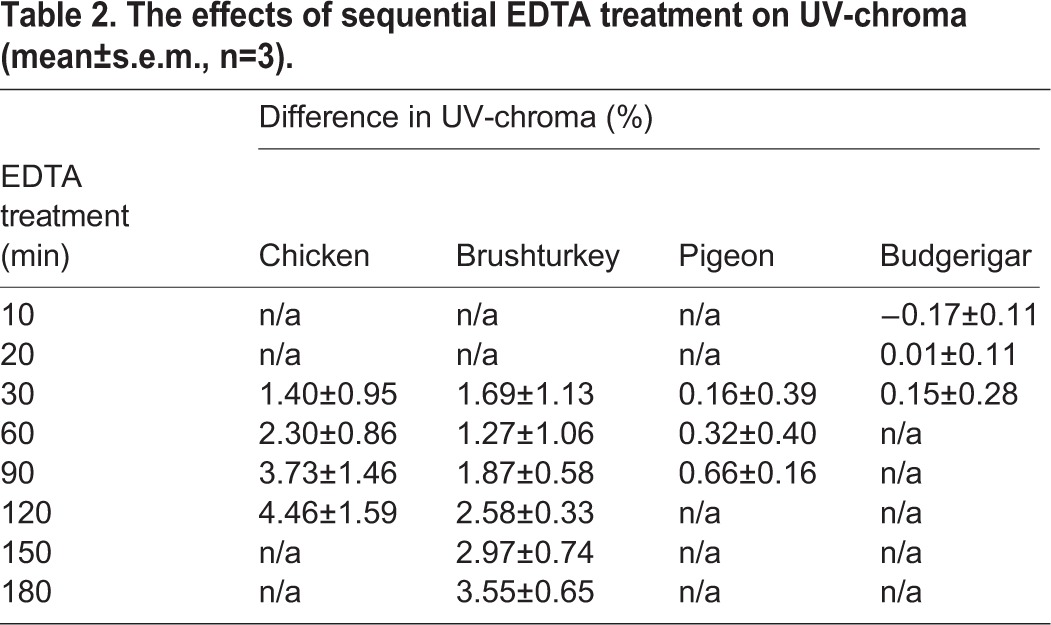
**The effects of sequential EDTA treatment on UV-chroma (mean±s.e.m., n=3).**

**Fig. 4.**
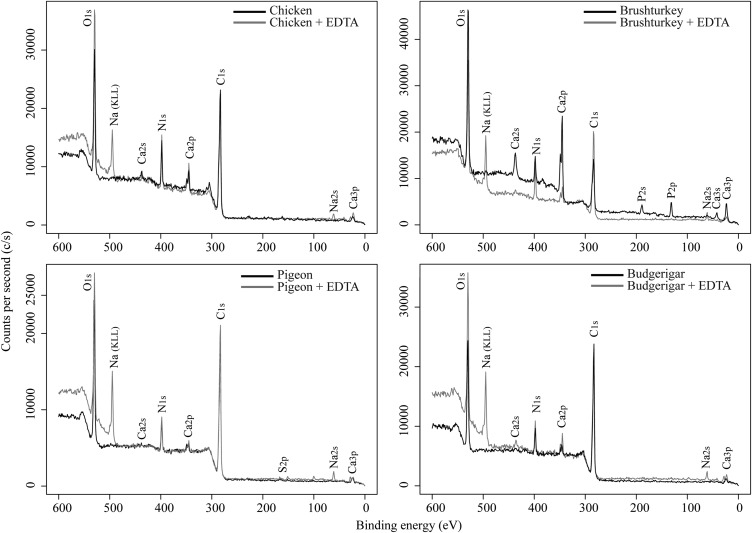
**XPS survey spectra showing the chemical composition of eggshells before and after EDTA treatment.** The EDTA treatment duration are 90 min for chicken, brushturkey, pigeon, and 30 min for budgerigar. The sodium peak results from the residual presence of EDTA, and was not taken into account to calculate the atomic percentages.

X-ray photoelectron spectroscopy (XPS) revealed the presence of phosphorus on the surface of chicken and brushturkey eggs, which completely disappeared following 90 min of EDTA treatment (Fig. 4; Table 3).

**Table 3.**
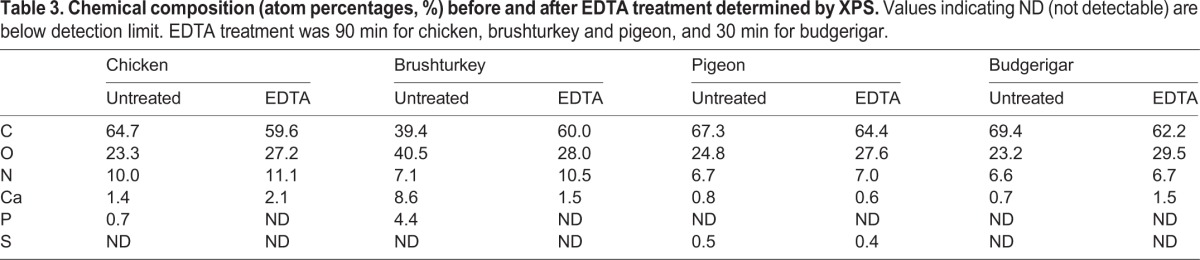
**Chemical composition (atom percentages, %) before and after EDTA treatment determined by XPS. Values indicating ND (not detectable) are below detection limit. EDTA treatment was 90 min for chicken, brushturkey and pigeon, and 30 min for budgerigar.**

## DISCUSSION

Despite the absence of known eggshell pigments (biliverdin and protoporphyrin), we found differences in the UV-reflectance of the four species’ eggshells. We showed that removal of the outer layers of avian eggshells that contain a cuticle increases UV-chroma, suggesting that the cuticle modulates UV-reflectance of white eggshells. This is likely achieved by selective absorption of UV-wavelengths by the compounds in the cuticle. The effects of the cuticle on eggshell coloration are particularly important, because the composition, thickness and extent of coverage of the cuticle (and thus potentially colour of the shell) can vary according to female age and egg freshness (Rodríguez-Navarro et al., 2013). These results highlight the importance of factors other than biliverdin and protoporphyrin in influencing avian eggshell coloration.

Eggshell colour varied across these unpigmented eggshells, and differed from that of pure calcite, even after their cuticles were removed (supplementary material Fig. S4). Although avian eggshells consist of approximately 96% calcite overall (Hamilton, 1986), the underlying structure of calcite crystals, or the composition of the organic matrix, can differ among species (Panheleux et al., 1999). These differences may cause variation in UV-chroma among the different species’ eggs studied here and highlight a role of non-pigmentary chemical or structural differences in influencing avian eggshell coloration. The chicken eggshell is particularly interesting as its UV-chroma drastically increased following removal of its outer layers. This finding suggests that some characteristic of the chicken eggshell increases the inherent UV-reflectance of calcite (supplementary material Fig. S4), possibly through nanostructuring as no identified pigment absorbs light across all wavelengths except UV (Andersson, 1999); however, the exact mechanism requires further investigation.

The increase in UV-chroma associated with removal of the outer eggshell layers was highest for eggshells with a clearly defined cuticle. EDTA treatment had the largest effect on chicken eggs, likely because it caused the greatest proportional decrease in eggshell thickness (Table 1), meaning that additional material other than the cuticle was removed. It is therefore possible that the drastic increase in UV-chroma is caused by interaction of light with structures or compounds inside the underlying palisade layer. By contrast, UV-chroma of budgerigar eggshells, which lack a cuticle (Mikhailov, 1997), did not increase after treatment. Despite the previously reported absence of cuticles on pigeon eggshells (Board, 1974), we found evidence of a very thin cuticle on one of the three pigeon eggshells (supplementary material Fig. S2), and it is likely that its removal caused the low (<1%), but significant, increase in UV-chroma. Indeed, it has been suggested that cuticles may be present on some freshly laid, open-nesting pigeon's eggs (Mikhailov, 1997). Our data thus suggest that the cuticle absorbs UV-light.

The composition of the cuticle varied between chicken and brushturkey, and EDTA treatment resulted in differential effects on eggshell thickness, making it difficult to identify the precise cause of the increase in UV-chroma. Unlike the mostly calcareous eggshell layer underneath, the XPS data showed the presence of phosphorous in the cuticles of chicken and brushturkey eggs (Table 3). This is likely coming from inorganic calcium phosphates, probably in the form of hydroxyapatite (Dennis et al., 1996; Board et al., 1984; D'Alba et al., 2014). Chicken cuticles mainly consists of proteins (85–90%), polysaccharides (4–5%), and lipids (2.5–3.5%) (Baker and Balch, 1962; Wedral et al., 1974; Hamilton, 1986; Rodríguez-Navarro et al., 2013). Therefore, these organic components may selectively absorb wavelengths in the UV-range (Holiday, 1936; Edelhoch, 1967; Itagaki, 1994; Albalasmeh et al., 2013). The small amount of inorganic phosphates may also selectively absorb UV-wavelengths (Holzmann et al., 2009; Piccirillo et al., 2014). The cuticle of brushturkey eggshells is composed predominantly of calcium phosphates (Board et al., 1984; D'Alba et al., 2014) and may have a similar effect on UV-absorbance.

The function of UV-reflectance by eggshells is unclear and needs more focal functional studies (Lahti, 2008) and broad comparative studies on eggshell composition and colour in relation to ecology (Cassey et al., 2012b). Substantial variation in ultraviolet coloration could alter the effectiveness of egg camouflage or UV protection, or impact mate choice. Whether variation in cuticle thickness or composition is sufficient to affect such changes are excellent topics for future research.

Avian eggshells are a good model system for inspiring biomimetic materials (Yoo et al., 2009). The modulation of UV-radiation is of prime importance for the design of many materials, including textiles, polymer coatings and paints (Andrady et al., 1998), because it can reduce detrimental effects of sun-exposure. UV-coloration produced through structural colour is likely less costly over the long-term than that produced using pigments because they are more durable (Sun et al., 2013), and thus more efficient for UV-protective coatings. Understanding the non-pigmentary mechanisms behind UV-modulation of avian eggshells could reveal potential new insights for the development of innovative UV-protective materials. In particular, unpigmented chicken eggshells are a prime candidate for further biomimetic study because their UV-reflectance characteristics are above that of calcite alone.

## MATERIAL AND METHODS

### Samples

We sourced three unincubated, untreated and non-pasteurized eggs of four species: chicken (*Gallus gallus*) eggs from a commercial farm in Akron, Ohio; Australian brushturkey (*Alectura lathami*) eggs from Brisbane, Australia; king pigeon (*Columba livia domestica*) eggs from a breeder in Dallas, TX; and budgerigar (*Melopsittacus undulatus*) eggs from a captive research colony in Las Cruces, NM. Eggshells were fragmented into 1 cm^2^ pieces using soft pressure and washed using 100% ethanol. We measured pigment concentration to verify the absence of biliverdin and protoporphyrin. We compared diffuse reflectance and conducted scanning electron microscopy (SEM) and X-ray photoelectron spectroscopy (XPS) on eggshells before and after chemical dissolution of the outer shell layers.

### Pigment extraction

We followed a modified pigment extraction protocol of Gorchein et al., (2009). We used the solvent alone as negative control, a brown chicken egg for protoporphyrin positive control and a blue chicken egg (Araucana strain) as biliverdin positive control. Briefly, shell samples were broken into small fragments (surface area ∼1 cm^2^ and/or weight ∼400 mg), rinsed with distilled water, 70% ethanol and homogenized by grinding; then 1 ml of aqueous solution of disodium ethylenediaminetetraacetic acid (EDTA) pH 7.2 (100 mg/ml) was added, and the tubes were vortex-mixed for 1 min and centrifuged at 15,000 ***g*** for 30 s in an Eppendorf 5430R Centrifuge, discarding the supernatants. This procedure was repeated three times and then 1 ml of acetonitrile-acetic acid (4:1 v/v) was added. The tubes were vortex-mixed for 2 min in 30 s bursts (and opened to allow the escape of CO_2_), and subsequently centrifuged for 2 min at 15,000 ***g***. The supernatants were then transferred to clean tubes and stored at 4°C in the dark until further analysis within 24 h. An aliquot was measured in a NanoDrop 2000c spectrophotometer for its UV-Vis absorbance spectrum from 250–700 nm versus acetonitrile-acetic acid as a blank. Pigment presence or absence was indicated from these spectra and confirmed and quantified by Ultra High Performance Liquid Chromatography (UHPLC) and Mass Spectrophotometry (MS). All shell extracts (whether or not pigment was detected by methods above) were further analysed through MS ion detection at specific masses (563 *m/z* for protoporphyrin and 583 *m/z* for biliverdin) to detect presence of pigments below the detection threshold of standard MS analysis. All observed pigments were also compared to commercially obtained standards of the free acids of biliverdin and protoporphyrin from Frontier Scientific Inc. (UT, USA) dissolved in acetonitrile-acetic acid.

### Experimental removal of outer layers

To experimentally investigate the contribution of the cuticle to the optical properties of the eggshells, we sequentially removed the outer eggshell layers (including the cuticle if present) over a course of treatments. For each treatment, we floated eggshells (with their surface down) on a weak alkaline solution (pH 8.1) of 0.37M EDTA and then gently brushed the surface using soft tissue paper (Baker and Balch, 1962; Igic et al., 2015). We repeated this over a course of treatment times depending on the thickness of the eggshells: successive increments of 10 min for budgerigar and increments of 30 min for chicken, brushturkey, and pigeon. We repeated treatments until the eggshells became too thin and fragile to handle (30 min for budgerigar, 90 min for pigeon, 120 min for chicken and 180 min for brushturkey). The removal of the outer layers was visualised by SEM after 30 and 90 min of EDTA treatment (or only after 30 min for the budgerigar).

### Scanning electron microscopy (SEM)

We mounted untreated and EDTA-treated eggshell fragments onto aluminium stubs, allowing the visualisation of both the shell surface and cross-section, which we then sputter-coated with gold/palladium for 3 min. SEM (JSM7401F, JEOL Japan) images were taken at a working distance of 8 mm with an accelerating voltage of 5 kV.

### Spectrophotometry

We measured diffuse reflectance on eggshell fragments between 300 and 700 nm. To minimize geometric variation associated with shell curvature and rough surfaces, we measured reflectance from the flattest part of fragments taken from the equatorial region of eggs. We used an integrating sphere (AvaSphere-50-REFL) with a black gloss trap to exclude specular reflectance, an AvaSpec-2048 spectrometer, and an AvaLight-XE pulsed xenon light source (Avantes Inc., Broomfield, CO, USA). All reflectance measurements were taken relative to a diffuse white standard (WS-2, Avantes Inc.).

We quantified UV-reflectance because this region showed the greatest level of variation for our samples. To evaluate changes in UV-reflectance, we calculated UV-chroma as a proportion of UV-reflectance from total reflectance (R_300-400_/R_300-700_) using the summary function of the R package PAVO (Maia et al., 2013). UV-chroma accounts for differences in total reflectance and thereby eliminates the confounding effect of eggshell thickness on our results. We then compared UV-chroma of eggshells across sequential EDTA treatments.

We used linear models to test if UV-chroma changed following sequential removal of the outer layers. For each species separately, we constructed models with UV-chroma as responses, egg ID as discrete predictor and EDTA treatment as continuous predictor. We constructed models using normal error distributions and identity link functions (supplementary material Table S1). We analysed each species separately because: (i) EDTA treatment durations were not quantitatively the same for the four species because of their differences in eggshell thickness and (ii) it was unclear whether EDTA treatment had the same effects for all other species’ eggs. P-values were adjusted following Holm's method (Aickin and Gensler, 1996). All statistical tests were implemented in R v.3.0.1 (R Development Core Team, 2013).

### X-ray photoelectron spectroscopy (XPS)

The survey spectra of untreated and EDTA-treated eggshells (90 min for chicken, brushturkey, and pigeon eggs; and 30 min for budgerigar eggs) were collected using a VersaProbe II Scanning XPS Microprobe from Physical Electronics (PHI), under ultrahigh vacuum conditions with a pressure of 2×10^−6^ Pa. Automated dual beam charge neutralization was used during the analysis of the samples to provide accurate data. The analyser pass energy was 117.4 eV and each spectrum was collected using a monochromatic Al Kα X-rays (hν=1486 eV) over a 200 μm diameter analysis area. The survey scans were used to evaluate the near surface region elemental composition of the eggshells. Peak areas were measured for the C 1s, O 1s, Ca 2p, N 1s, P 2p and S 2p regions and elements were quantified using instrument-modified Schofield cross sections (PHI MultiPak software). The sodium peak results from the residual presence of EDTA, and was not taken into account to calculate the atomic percentages. Under ideal conditions, this technique allows the detection of elements that have near surface region concentrations higher than ∼1% by weight at an analysis depth of approximately 10 nm. However, surface roughness can affect quantification accuracy.

Governmental and institutional guidelines were followed in sourcing and using biological materials.

## Supplementary Material

Supplementary Material

## References

[BIO012211C1] Aickin M. and Gensler H. (1996). Adjusting for multiple testing when reporting research results: the Bonferroni vs Holm methods. *Am. J. Public Health* 86, 726-728. 10.2105/AJPH.86.5.726 8629727 PMC1380484

[BIO012211C2] Albalasmeh A. A., Berhe A. A. and Ghezzehei T. A. (2013). A new method for rapid determination of carbohydrate and total carbon concentrations using UV spectrophotometry. *Carbohydr. Polym.* 97, 253-261. 10.1016/j.carbpol.2013.04.072 23911443

[BIO012211C3] Andersson S. (1999). Morphology of UV reflectance in a whistling-thrush: implications for the study of structural colour signalling in birds. *J. Avian Biol.* 30, 193-204. 10.2307/3677129

[BIO012211C4] Andrady A. L., Hamid S. H., Hu X. and Torikai A. (1998). Effects of increased solar ultraviolet radiation on materials. *J. Photochem. Photobiol. B* 46, 96-103. 10.1016/S1011-1344(98)00188-2 9894353

[BIO012211C5] Baker J. R. and Balch D. A. (1962). A study of the organic material of hen's-egg shell. *Biochem. J.* 82, 352-361. 13864145 10.1042/bj0820352 PMC1243460

[BIO012211C48] Board R. G. (1974). Microstructure, water resistance and water repellency of the pigeon egg shell. *Br. Poult. Sci.* 15, 415-419. 10.1080/00071667408416126 4414675

[BIO012211C6] Board R. G., Perrott H. R., Love G. and Scott V. D. (1984). The phosphate-rich cover on the eggshells of grebes (Aves: Podicipitiformes). *J. Zool.* 203, 329-343. 10.1111/j.1469-7998.1984.tb02336.x

[BIO012211C7] Bogrekci I. and Lee W. S. (2004). Spectral signatures of common phosphates in soils and their effect on absorbance spectra of soil samples with different phosphorus concentrations. In *Proceedings of the Annual International Meeting of American Society of Agricultural Engineers and Canadian Society of Agricultural Engineers, Ottawa, ON, Canada*. Meeting Paper No. 043114 St Joseph, MI: ASAE.

[BIO012211C8] Bonderer L. J., Studart A. R. and Gauckler L. J. (2008). Bioinspired design and assembly of platelet reinforced polymer films. *Science* 319, 1069-1073. 10.1126/science.1148726 18292337

[BIO012211C9] Cassey P., Mikšík I., Portugal S. J., Maurer G., Ewen J. G., Zarate E., Sewell M. A., Karadas F., Grim T. and Hauber M. E. (2012a). Avian eggshell pigments are not consistently correlated with colour measurements or egg constituents in two Turdus thrushes. *J. Avian Biol.* 43, 503-512. 10.1111/j.1600-048X.2012.05576.x

[BIO012211C10] Cassey P., Thomas G. H., Portugal S. J., Maurer G., Hauber M. E., Grim T., Lovell P. G. and Mikšík I. (2012b). Why are birds’ eggs colourful? Eggshell pigments co-vary with life-history and nesting ecology among British breeding non-passerine birds. *Biol. J. Linn. Soc.* 106, 657-672. 10.1111/j.1095-8312.2012.01877.x

[BIO012211C11] D'Alba L., Jones D. N., Badawy H. T., Eliason C. M. and Shawkey M. D. (2014). Antimicrobial properties of a nanostructured eggshell from a compost-nesting bird. *J. Exp. Biol.* 217, 1116-1121. 10.1242/jeb.098343 24311808

[BIO012211C12] Dennis J. E., Xiao S.-Q., Agarwal M., Fink D. J., Heuer A. H. and Caplan A. I. (1996). Microstructure of matrix and mineral components of eggshells from White Leghorn chickens (Gallus gallus). *J. Morphol.* 228, 287-306. 10.1002/(SICI)1097-4687(199606)228:3<287::AID-JMOR2>3.0.CO;2-# 29852681

[BIO012211C13] Edelhoch H. (1967). Spectroscopic determination of tryptophan and tyrosine in proteins. *Biochemistry* 6, 1948-1954. 10.1021/bi00859a010 6049437

[BIO012211C14] Finnemore A., Cunha P., Shean T., Vignolini S., Guldin S., Oyen M. and Steiner U. (2012). Biomimetic layer-by-layer assembly of artificial nacre. *Nat. Commun.* 3, 966 10.1038/ncomms1970 22828626

[BIO012211C15] Gorchein A., Lim C. K. and Cassey P. (2009). Extraction and analysis of colourful eggshell pigments using HPLC and HPLC/electrospray ionization tandem mass spectrometry. *Biomed. Chromatogr.* 23, 602-606. 10.1002/bmc.1158 19277957

[BIO012211C16] Grégoire C. (1957). Topography of the organic components in mother-of pearl. *J. Biophys. Biochem. Cytol.* 3, 797-808. 10.1083/jcb.3.5.797 13475393 PMC2224113

[BIO012211C17] Hamilton R. M. G. (1986). The microstructure of the hen's egg shell – a short review. *Food Microstruct.* 5, 99-110.

[BIO012211C18] Hauber M. E. (2014). The Book of Eggs: A Life-Size Guide to the Eggs of Six Hundred of the World's Bird Species. Chicago, IL: University of Chicago Press.

[BIO012211C19] Hill G. E. and McGraw K. J. (2006). *Bird Coloration: Function and Evolution*. Cambridge, MA: Harvard University Press.

[BIO012211C20] Hincke M. T., Bernard A. M., Lee E. R., Tsang C. P. W. and Narbaitz R. (1992). Soluble protein constituents of the domestic fowl's eggshell. *Br. Poult. Sci.* 33, 505-516. 10.1080/00071669208417489 1643516

[BIO012211C21] Holiday E. R. (1936). Spectrophotometry of proteins: absorption spectra of tyrosine, tryptophan and their mixtures. II. Estimation of tyrosine and tryptophan in proteins. *Biochem. J.* 30, 1795-1803. 16746224 10.1042/bj0301795 PMC1263262

[BIO012211C22] Holzmann D., Holzinger D., Hesser G., Schmidt T. and Knör G. (2009). Hydroxyapatite nanoparticles as novel low-refractive index additives for the long-term UV-photoprotection of transparent composite materials. *J. Mater. Chem.* 19, 8102-8106. 10.1039/b912116a

[BIO012211C23] Igic B., Cassey P., Grim T., Greenwood D. R., Moskát C., Rutila J. and Hauber M. E. (2012). A shared chemical basis of avian host-parasite egg colour mimicry. *Proc. Biol. Sci.* 279, 1068-1076. 10.1098/rspb.2011.1718 21920975 PMC3267149

[BIO012211C24] Igic B., Fecheyr-Lippens D., Xiao M., Chan A., Hanley D., Brennan P. R. L., Grim T., Waterhouse G. I. N., Hauber M. E. and Shawkey M. D. (2015). A nanostructural basis for gloss of avian eggshells. *Proc. R. Soc. Lond. B* 12, 20141210 10.1098/rsif.2014.1210 PMC4305423 25505139

[BIO012211C25] Itagaki H. (1994). Saccharification process of cellulose in 97% sulfuric acid monitored by sulfuric acid induced ultraviolet absorption behaviour. *Polymer* 35, 50-52. 10.1016/0032-3861(94)90048-5

[BIO012211C26] Kennedy G. Y. and Vevers H. G. (1976). A survey of avian eggshell pigments. *Comp. Biochem. Physiol.* 55B, 117-123. 10.1016/0305-0491(76)90183-8 947658

[BIO012211C27] Kilner R. M. (2006). The evolution of egg colour and patterning in birds. *Biol. Rev. Camb. Philos. Soc.* 81, 383-406. 10.1017/S1464793106007044 16740199

[BIO012211C28] Kinoshita S., Yoshioka S. and Miyazaki J. (2008). Physics of structural colors. *Rep. Prog. Phys.* 71, 076401 10.1088/0034-4885/71/7/076401

[BIO012211C29] Kusuda S., Iwasawa A., Doi O., Ohya Y. and Yoshizaki N. (2011). Diversity of the cuticle layer of avian eggshells. *J. Poult. Sci.* 48, 119-124. 10.2141/jpsa.010103

[BIO012211C30] Lahti D. C. (2008). Population differentiation and rapid evolution of egg color in accordance with solar radiation. *Auk* 125, 796-802. 10.1525/auk.2008.07033

[BIO012211C31] Li Q., Xia L., Zhang Z. and Zhang M. (2010). Ultraviolet extinction and visible transparency by ivy nanoparticles. *Nanoscale Res. Lett.* 5, 1487-1491. 10.1007/s11671-010-9666-2 20730120 PMC2920404

[BIO012211C32] Maia R., Eliason C. M., Bitton P.-P., Doucet S. M. and Shawkey M. D. (2013). pavo: an R package for the analysis, visualization and organization of spectral data. *Methods Ecol. Evol.* 4, 906-913. 10.1111/2041-210X.12069

[BIO012211C33] Maurer G., Portugal S. J., Hauber M. E., Mikšík I., Russell D. G. D. and Cassey P. (2015). First light for avian embryos: eggshell thickness and pigmentation mediate variation in development and UV exposure in wild bird eggs. *Funct. Ecol.* 29, 209-218. 10.1111/1365-2435.12314

[BIO012211C34] Merilaita S. and Lind J. (2005). Background-matching and disruptive coloration, and the evolution of cryptic coloration. *Proc. R. Soc. Lond. B* 272, 665-670. 10.1098/rspb.2004.3000 PMC1564081 15817442

[BIO012211C35] Mikhailov K. E. (1997). Avian Eggshells: an Atlas of Scanning Electron Micrographs. Peterborough: British Ornithologists’ Club.

[BIO012211C36] Moreno J. and Osorno J. L. (2003). Avian egg colour and sexual selection: does eggshell pigmentation reflect female condition and genetic quality? *Ecol. Lett.* 6, 803-806. 10.1046/j.1461-0248.2003.00505.x

[BIO012211C37] Panheleux M., Bain M., Fernandez M. S., Morales I., Gautron J., Arias J. L., Solomon S. E., Hincke M. and Nys Y. (1999). Organic matrix composition and ultrastructure of eggshell: a comparative study. *Br. Poult. Sci.* 40, 240-252. 10.1080/00071669987665 10465392

[BIO012211C38] Parker A. R. (2000). 515 million years of structural colour. *J. Opt. Pure Appl. Opt.* 2, R15-R28. 10.1088/1464-4258/2/6/201

[BIO012211C39] Piccirillo C., Rocha C., Tobaldi D. M., Pullar R. C., Labrincha J. A., Ferreira M. O., Castro P. M. L. and Pintado M. M. E. (2014). A hydroxyapatite–Fe_2_O_3_ based material of natural origin as an active sunscreen filter. *J. Mater. Chem. B* 2, 5999-6009. 10.1039/C4TB00984C 32261852

[BIO012211C40] R Development Core Team (2013). *R: A Language and Environment for Statistical Computing.* R Foundation for Statistical Computing, Vienna, Austria. ISBN 3-900051-07-0 Available at: http://www.R-project.org.

[BIO012211C41] Rodríguez-Navarro A. B., Domínguez-Gasca N., Muñoz A. and Ortega-Huertas M. (2013). Change in the chicken eggshell cuticle with hen age and egg freshness. *Poult. Sci.* 92, 3026-3035. 10.3382/ps.2013-03230 24135608

[BIO012211C42] Sparks N. H. C. (1994). Shell accessory materials: structure and function. In *Microbiology of the Avian Egg* (Board R. G. and Fuller R.), pp. 25-42. New York, NY: Springer.

[BIO012211C43] Srinivasarao M. (1999). Nano-optics in the biological world: beetles, butterflies, birds, and moths. *Chem. Rev.* 99, 1935-1962. 10.1021/cr970080y 11849015

[BIO012211C44] Sun J., Bhushan B. and Tong J. (2013). Structural coloration in nature. *RSC Advances* 3, 14862-14889. 10.1039/c3ra41096j

[BIO012211C45] Wedral E. M., Vadehra D. V. and Baker R. C. (1974). Chemical composition of the cuticle, and the inner and outer shell membranes from eggs of Gallus gallus. *Comp. Biochem. Physiol.* 47B, 631-640. 10.1016/0305-0491(74)90011-X 4217681

[BIO012211C46] Yang C., Wang L., Hsu Y.-C., Antonov A., Moksnes A., Røskaft E., Liang W. and Stokke B. G. (2013). UV reflectance as a cue in egg discrimination in two Prinia species exploited differently by brood parasites in Taiwan. *Ibis* 155, 571-575. 10.1111/ibi.12043

[BIO012211C47] Yoo S., Hsieh J. S., Zou P. and Kokoszka J. (2009). Utilization of calcium carbonate particles from eggshell waste as coating pigments for ink-jet printing paper. *Bioresour. Technol.* 100, 6416-6421. 10.1016/j.biortech.2009.06.112 19665373

