# Supplementary material for: The cuticle modulates ultraviolet reflectance of avian eggshells
Source: Biol Open. 2015 May 11;4(7):753–9. doi: 10.1242/bio.012211 (PMC4571098; doi:10.1242/bio.012211)
Supplement: Supplementary Material [file supp_4_7_753__index.html]

The cuticle modulates ultraviolet reflectance of avian eggshells — The cuticle modulates ultraviolet reflectance of avian eggshells — Supplementary Material 

# The cuticle modulates ultraviolet reflectance of avian eggshells

## BIO012211 Supplementary Material

- Supplementary Material
