# Supplementary material for: The cuticle modulates ultraviolet reflectance of avian eggshells
Source: Biol Open. 2015 May 11;4(7):753–9. doi: 10.1242/bio.012211 (PMC4571098; doi:10.1242/bio.012211)
Supplement: Supplementary Material [file supp_bio.012211_BIO012211supp.pdf]

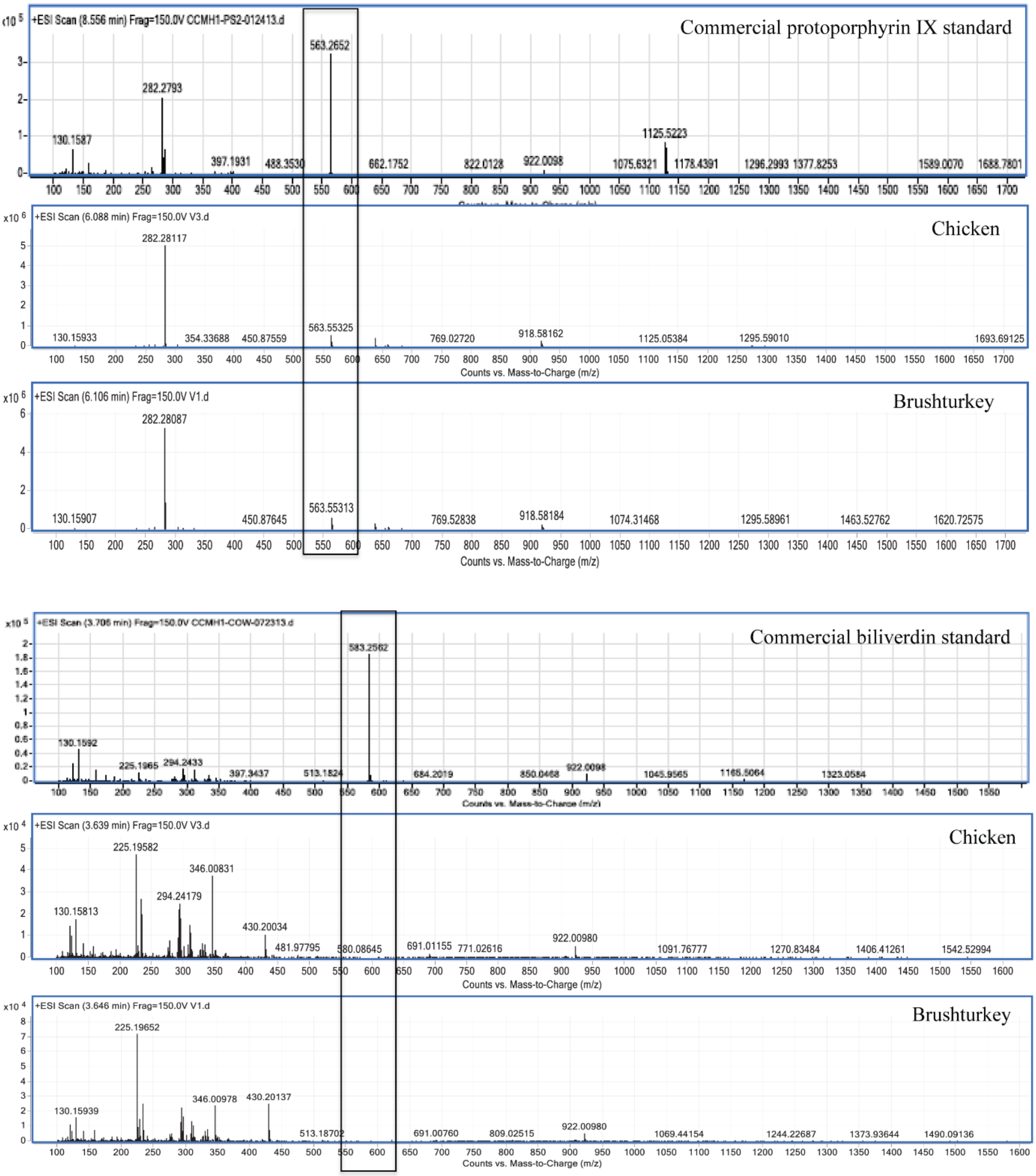

Supplementary material Fig. S1. Mass spectra of chicken and brushturkey eggshell extracts are shown as example of eggs that lack a detectable amount of protoporphyrin (upper three) and biliverdin (lower three).

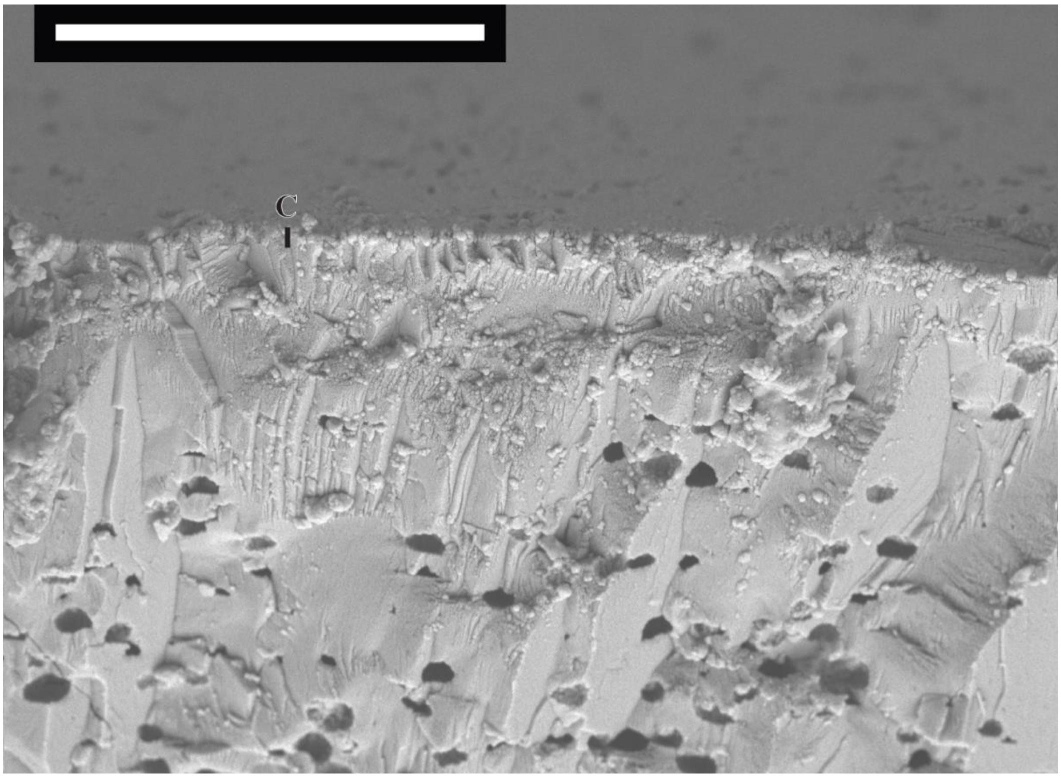

Supplementary material Fig. S2. Cross-sectional SEM image of one particular pigeon egg showing a structure resembling a very thin cuticle (C). Scale bar is 10 µm.

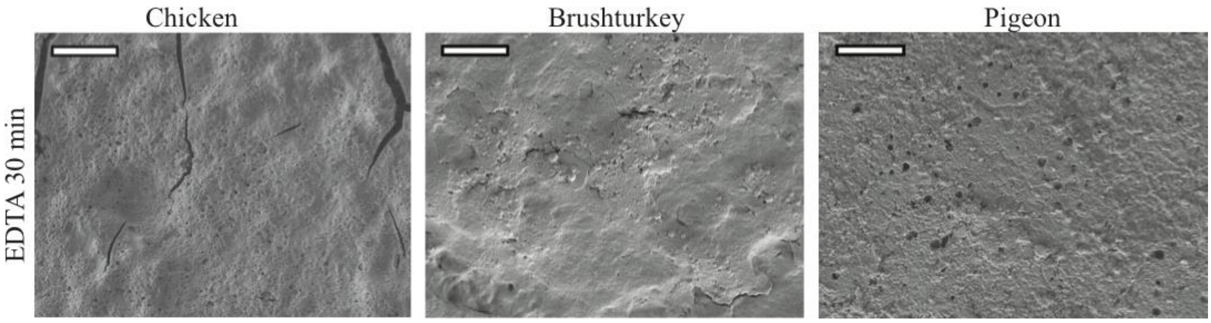

Supplementary material Fig. S3. Effect of 30 min EDTA treatment on the surface morphologies of chicken, brushturkey and pigeon.

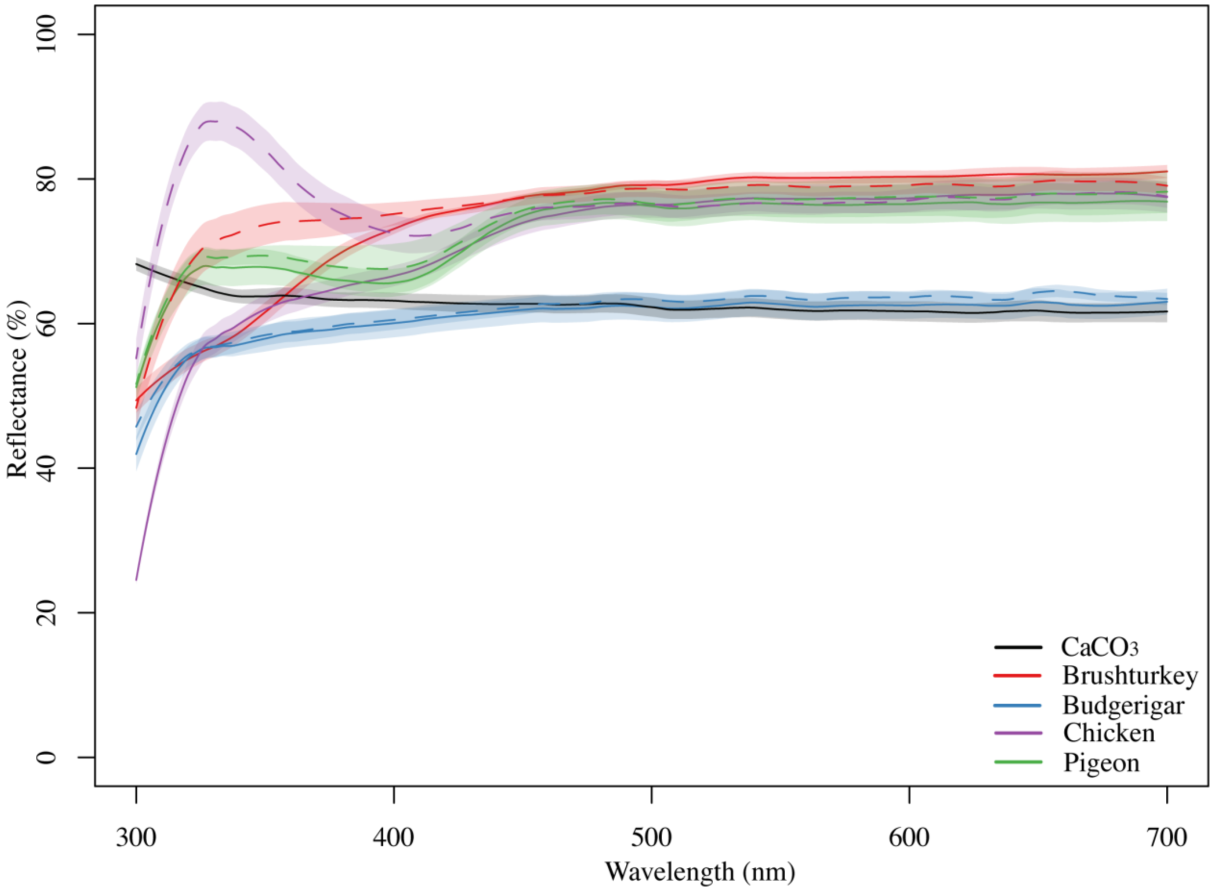

Supplementary material Fig. S4. Diffuse reflectance of a thin, flat layer of pure calcite powder (Sigma Aldrich, St. Louis, MO, USA), compared to those of untreated (solid lines) and EDTA-treated (dashed lines) eggshells.

Table S1. Summary output for linear models comparing the change in UV chroma in relation to EDTA treatment.

Species	UV chroma[i]	95 % C.I.	Term[ii]	*F*	dfs	*P*[iii]
Chicken	3.92e-02 ± 0.38e-02	[3.07e-04, 4.77e-04]	Egg ID	3.33	2, 11	0.074
			EDTA	103.66	1, 11	< 0.001
Brushturkey	1.73e-02 ± 0.22e-02	[1.27e-04, 2.19e-04]	Egg ID	5.93	2, 17	0.011
			EDTA	61.95	1, 17	< 0.001
Pigeon	0.71e-02 ± 0.21-02	[0.23e-04, 1.20e-04]	Egg ID	5.43	2, 8	0.032
			EDTA	11.62	1, 8	0.009
Budgerigar	0.62e-02 ± 0.47-02	[-0.45e-04, 1.70e-04]	Egg ID	1.09	2, 8	0.380
			EDTA	1.78	1, 8	0.219

[i]Change in UV chroma (%) per min of EDTA treatment ± SE

[ii]Egg ID: number of egg; EDTA: time of EDTA treatment

[iii]P-values for EDTA were adjusted following Holm’s method
